# Mechanical Properties and Electromagnetic Interference Shielding of Carbon Composites with Polycarbonate and Acrylonitrile Butadiene Styrene Resins

**DOI:** 10.3390/polym15040863

**Published:** 2023-02-09

**Authors:** Jeong Keun Kim, Sung-Soo Kang, Hong Gun Kim, Lee Ku Kwac

**Affiliations:** 1 Department of Carbon Convergence Engineering, Jeonju University, Jeonju-si 55069, Jeonbuk, Republic of Korea; 2 Department of Mechanical and Automotive Engineering, Jeonju University, Jeonju-si 55069, Jeonbuk, Republic of Korea

**Keywords:** tensile strength, flexural strength, impact strength, EMI shielding effectiveness, thermoplastics, carbon fiber, carbon nanotube

## Abstract

As environmental pollution becomes a serious concern, considerable effort has been undertaken to develop power devices with minimal production of carbon dioxide (CO_2_) and exhaust gases. Owing to this effort, interest in technologies related to hybrid and electric products that use fuel cells has been increasing. The risk of human injuries owing to electromagnetic waves generated by electrical and electronic devices has been also rising, prompting the development of mitigating technologies. In addition, antistatic devices for protecting operators from static electricity have also been considered. Therefore, in this study, we investigated the development of thermoplastic carbon composites containing carbon fibers (CFs) and carbon nanotubes (CNTs). Ultimately, materials with improved mechanical properties (e.g., flexural, impact, and tensile strength properties of about +61.09%, +21.44%, +63.56%, respectively), electromagnetic interference (EMI) shielding (+70.73 dB), and surface resistivity (nearly zero) can be developed by impregnating CFs and CNTs with polycarbonate (PC) and acrylonitrile butadiene styrene (ABS) resins, respectively. The total average mechanical properties of PC and ABS composites increased by 24.35% compared with that of ABS composites, while that of PC composites increased by 32.86% with that of PC and ABS composites. Therefore, in this study, we aimed to develop carbon composites, to take advantage of these thermoplastic resins.

## 1. Introduction

Polymers are widely used in various critical structures such as aircraft, automobiles, and pressure vessels [1,2,3]. Compared with metals, a smaller number of experimental and theoretical studies have addressed polymers [4].

With recent advancements in electromagnetic interference (EMI) shielding technologies and the rapid expansion of applications of electronic components in the automobile industry, electromagnetic wave bands have been gradually shifting toward higher frequencies. The incidence of electromagnetic pollution has been steadily increasing. Electromagnetic waves can penetrate into nearby electronic devices and can negatively affect them, or can cause them to malfunction. With the increasing number of electrically controlled automobile parts, safety accidents, such as malfunctioning and/or sudden acceleration of vehicles owing to electromagnetic waves, will likely become a major problem. Because electromagnetic waves can harm humans, effective EMI shielding technologies have been garnering increasing attention. Equipment that uses many high-density and precision parts is quite susceptible to EMI; thus, this interference can significantly affect modern electric and electronic telecommunication industries that seek to develop miniature, highly integrated, high-speed, and multifunctional devices [5]. In comparison with conventional metal products, electrical conducting polymer products can be light in weight, and have resistance to corrosion, easier processing, and flexibility, as found by Micheli et al. [6]. In particular, the use of nanofillers such as CNFs results in better absorption performance than do conventional graphite absorbers, which typically require higher thicknesses to obtain similar absorption properties. For this reason, radar absorbing materials (RAM) have importance in those products requiring microwave absorption with low mass and volume.

Existing EMI shielding technologies mainly use metals in their base or in coatings of conductive films, or they utilize plating technologies. However, with the increasingly complex patterning of metal products, their processability worsens and they become increasingly difficult to fabricate [7]. On the other hand, because polymer composites can be commercialized using injection processes, their advantages in terms of production cost and efficiency are considerable. In addition, polymer composites are advantageous owing to their low density, durability, and excellent moldability [8]. Polymer composites with good EMI shielding performance are light and flexible compared with metals, and are not corroded by conductive fillers that are often added to polymer resins [9]. Because their physical properties are easy to control, these polymers have been widely used as materials for EMI shielding. Among the various conductive fillers, carbon materials have a low specific gravity and excellent electrical properties; thus, they have been very promising as EMI shielding materials [10].

One efficient way to enhance EMI shielding is to blend with different polymers, especially thermoplastics. Consequently, blends of polycarbonate (PC) and acrylonitrile butadiene styrene (ABS) have been broadly utilized in engineering applications [4]. Varying the proportion of the polymers enables controlling the blends’ characteristics and properties. Desired properties can be obtained by blending and adding other reinforcing materials, such as carbon fibers (CFs) and carbon nanotubes (CNTs) [11]. For example, PC is known for its excellent mechanical properties, such as tensile, flexural, and impact strength. Although it is durable, it is vulnerable to scratches and abrasion, and it is sensitive to organic solvents such as alkaline detergents, acetone, and benzene; therefore, its chemical resistance is low [12]. ABS is easy to process and exhibits strong impact and heat resistance; therefore, it is often used as a metal substitute for interior and exterior materials in automobiles. However, owing to a lack of transparency and weather resistance, additives are required for addressing these drawbacks [7].

The components of PC and ABS alloys have economically complementary properties. They have garnered attention in engineering applications such as automotive engineering, owing to their toughness. Therefore, research on these alloys is important for determining the scope of their engineering applications, and many studies have been conducted. In particular, their tensile deformation and toughness were examined [13].

PC and ABS have similar polarities, making them potentially compatible with each other. ABS is firmly bonded by its styrene-acrylonitrile side chains, yielding better physical properties [14]. Under this fundamental assumption, it would be interesting to determine the dependence of the impact strength and modulus on the PC content. Because ABS is a mixture of thermoplastic styrene-acrylonitrile (SAN) and dispersed rubber butadiene particles, PC and ABS blends are referred to as ternary ones [4].

Because of their critical importance, PC and ABS blends have been widely analyzed in many studies; however, theoretical investigations are rare. Studies have shown that the mechanical properties of PC and ABS blends improve with increasing PC content [11].

The objective of this study was to compare the tensile strength, impact strength, flexural strength, electrical conductivity, and EMI shielding effectiveness of different blends. In this study, constant ratios of PC and ABS were used for obtaining specimens, and the results were compared and analyzed for blends with PC and ABS ratios of 100:0, 50:50, and 0:100. In the actual process of the experiment, we tried to apply CF and CNT, which are carbon materials, to polypropylene (PP) and polyethylene (PE) resins, not PC or ABS. The network between them was so weak that they couldn’t combine themselves. In order to improve this, an oxidizing agent was applied and mixed in an internal mixer in the same way, but it could not prevent the phenomenon of breaking and browning by itself. Accordingly, PC and ABS resins were used, which can be mixed in the standard internal mixer and have an excellent network between resins.

In order to compensate for the disadvantages of these PE or PP, several pellets were secured and additional experiments were conducted, but no improvement was found. When determining the content of carbon fiber, up to 30 wt% was tried, but using this mixture, an overload occurred in the experiment’s internal mixer and a malfunction occurred. Accordingly, it was decided to add up to 25 wt% of carbon fiber. Of course, it had been determined that the mechanical properties were relatively improved when 30 wt% of the carbon fiber was added. However, the present experimental conditions were set by the determination that the 25 wt% content was appropriate to prevent malfunction of the internal mixer equipment and sufficient to maximize mechanical strength.

In the case of carbon nanotubes, dispersibility is considered to be the main point when mixing with PC or ABS resin, and sufficient mixing time was set to improve this in this study. However, it is known that there is a limit to securing the dispersibility of carbon nanotubes in an internal mixer that is mixed by a physical method. In order to overcome these limitations, it will be necessary to find several methods to secure the dispersibility of carbon nanotubes. In previous studies, PC and ABS were impregnated with carbon materials to measure tensile strength, impact strength, flexural strength, electrical conductivity and electromagnetic shielding, but in this study, PC and ABS were mixed together in the additional experiments to compare the results. We tried to identify and review the differences between the cases. In the process of mixing PC and ABS with CF and CNT, the latter of which are carbon materials, in an internal mixer, efforts were made to find the RPM or temperature conditions, and to set the optimal RPM and temperature conditions to manufacture an ideal thermoplastic carbon composite. In the course of the experiment, some toxic gas was generated from thermoplastics such as PC and ABS, so the experiment was conducted with the utmost care wearing a safety mask and focusing on ventilation to the outside.

The hot heat generated from the internal mixer was a bit difficult to work with, and after using the internal mixer, PC and ABS resin remaining inside were removed as much as possible so that there were no impurities that could occur when new specimens were made. In addition, we did our best to complete the mixing process within 20 min so that the PC and ABS resin inside the internal mixer did not cause internal defects of the resin due to the hot temperature. When taking out the mixed sample from the internal mixer, the ABS composite was much easier than the PC composite; when taking out the PC composite, it was difficult to take it out due to its low viscosity. Afterwards, the sample taken out of the internal mixer was put into a hot press and compressed to fit the inside size of the jig. In order to compensate for this, after compressing with a hot press as much as possible, we waited until it had cooled naturally, rather than taking it out immediately, and then we took out the sample when the temperature had dropped to room temperature, as a sufficient time had elapsed.

After the sample was taken out after a sufficient time, the phenomenon of bending in the shape of the specimen disappeared. Originally, this study had been conducted to apply to the cover used for the exterior of the electric excavator. The cover blocks electromagnetic waves that may affect the electric excavator operator and uses a relatively easy-to-process thermoplastic resin for the outer cover, which is difficult to process with metal such as steel. The purpose was to reduce weight and improve mechanical properties. Therefore, through this study, we tried to reduce the weight by applying thermoplastics, which are lighter than metals, and improve the disadvantages of existing metals or thermosetting resins. The research objective was to improve the mechanical properties, EMI shielding effectiveness, and antistatic performence [15]. Through this study, we aimed to manufacture PC and ABS composites that combine the advantages of mechanical strength and transparency of PC composites with the easy processability of ABS composites, and by impregnating carbon materials of CF and CNT into thermoplastic resins, we also aimed to manufacture thermoplastic carbon composites with improved mechanical strength, electromagnetic wave shielding, and antistatic performance. Therefore, in the future, it is expected that it will be possible to manufacture carbon composites with mechanical strength and shielding performance using thermoplastic resins, which have better processability than thermosetting resins.

## 2. Experimental Investigation

### Preparation of Test Specimens

A total of 100 g of thermoplastic resin pellets (PC, ABS pellets), CFs (0, 5, 15, and 25 wt%), and CNTs (0, 2 wt%) were mixed and prepared for each content. An internal mixer (Teng Co., Ltd., Deajeon, Republic of Korea) was used to manufacture the thermoplastic composites, and the applied thermoplastic PC and ABS resins were Trirex 3025U (Sam-Yang, Inc., Seoul, Republic of Korea) and HF380 (LG Co., Ltd., Seoul, Republic of Korea), respectively. CFs were chopped carbon-6 mm (Length: 6 mm, Non-Sizing, Motionfive Co., Ltd., Goyang, Republic of Korea, Thickness: 5–7 μm, Aspect Ratio: 857–1200), and multi-walled CNTs (MWCNTs) were TMC100-10 (Nanosolution Co., Ltd., Jeonju, Republic of Korea). At the beginning of the experiment, the temperature of the internal mixer was set (PC: 240 °C, ABS: 200 °C) and when it rose to the appropriate temperature, the resins (PC or ABS) and reinforcements (chopped CFs, CNTs) were mixed and placed in the internal mixer. The speed of the internal mixer was approximately 5–6 rpm. For higher speeds, the mixing did not proceed smoothly, overloading the machine.

The samples were mixed as shown in Table 1 for approximately 20 min (in the case of chopped CF, they were sufficiently mixed with the resin for approximately 20 min), then taken out from the internal mixer, placed in the steel mold, the temperature of the hot press was set as shown in Table 2 (PC and ABS specimens: 230 °C, PC specimens:230 °C, ABS specimens: 180 °C), and the samples were then compressed at a pressure of 40 MPa. When the compression was judged to be sufficient (after 1 or 2 min), the hot press was lowered, and the specimens were folded. Again, at the molding temperature, the specimens were placed into the hot press together with the steel mold and compressed.

The process was repeated 3–4 times, ensuring no voids remained in the obtained specimens. The specimens were removed after the hot press cooled down to approximately 50–60 °C (PC and ABS: 50 °C, PC: 50 °C, ABS: 60 °C). PC specimens exhibited better bending properties after cooling than did ABS specimens; thus, the specimens were taken out at a lower temperature from the hot press. In addition, a natural cooling method was used while keeping the specimens in the hot press. Because the cooling rate tends to be excessively high during natural cooling at room temperature, air cooling was performed using a fan. After preparing the specimens, the mechanical properties (tensile, flexural, and impact strength characteristics), EMI shielding effectiveness, and surface resistivity (antistatic performance) were assessed according to each standard.

## 3. Results and Discussion

### 3.1. Comparison of Flexural Strength

**Figure 1:**
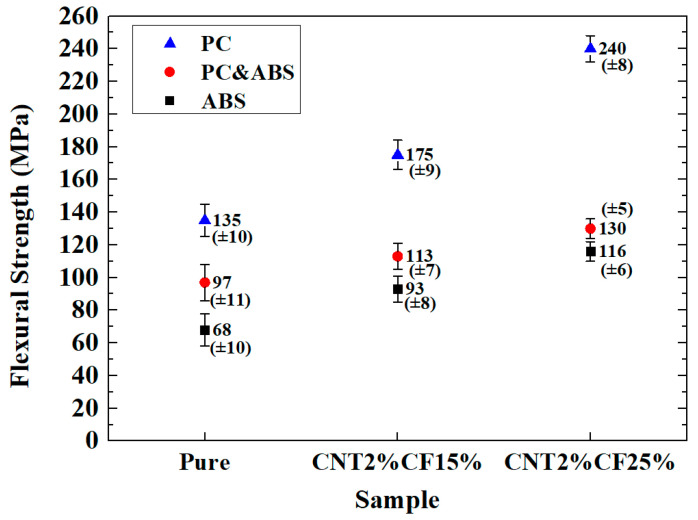
Comparison of flexural strength.

Figure 1 shows the comparison of flexural strengths. The prepared PC composites had the highest strength, the ABS composites exhibited the lowest strength, and the PC and ABS composites exhibited intermediate strength. The flexural strength of the PC and ABS specimens was, on average, 24.85% higher compared with the ABS specimens, while that of the PC specimens was 60.28% higher compared with the PC and ABS specimens. As the CF content increased, the flexural strength also increased. CNT2%/CF15% increased by 28.13% on average compared with pure specimens, while CNT2%/CF25% increased by 25.37% compared with CNT2%/CF15%.

### 3.2. Comparison of Impact Strength

**Figure 2:**
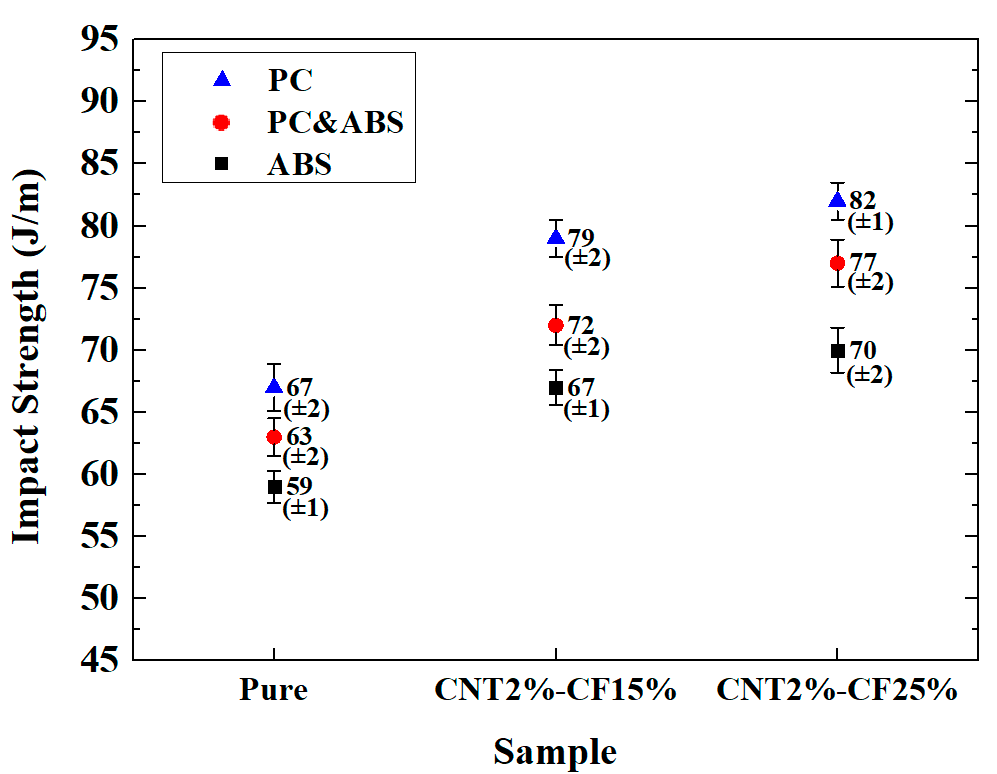
Comparison of impact strength.

Figure 2 compares the impact strengths of the specimens. The prepared PC composites had the highest strength, and the ABS composites exhibited the lowest strength, while the PC and ABS composites exhibited intermediate strength. The impact strength of the PC and ABS composites increased by 7.68% on average, compared with the ABS composites, while that of the PC specimens increased by 7.58% compared with the PC and ABS specimens. As the CF content increased, the impact strength also increased. CNT2%/CF15% increased by 15.37% on average compared with pure specimens, while CNT2%/CF25% increased by 5.28% compared with CNT2%/CF15%.

### 3.3. Comparison of Tensile Strength

**Figure 3:**
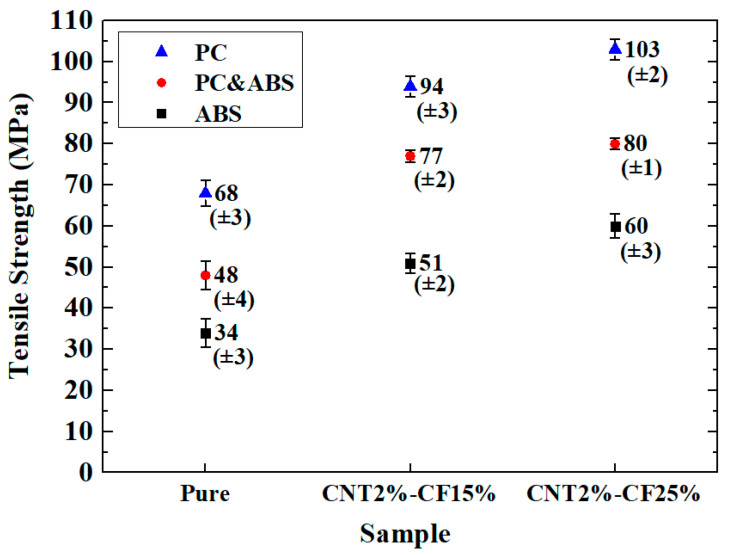
Comparison of tensile strength.

Figure 3 compares the tensile strengths. The prepared PC composites had the highest strength, the ABS composites exhibited the lowest strength, and the PC and ABS composites exhibited intermediate strength. The tensile strength of the PC and ABS composites increased by 40.52% on average compared with that of the ABS composites, while the strength of the PC specimens increased by 30.73% compared with that of the PC and ABS specimens. As the CF content increased, the tensile strength also increased. CNT2%/CF15% increased by 49.16% on average compared with pure specimens, while CNT2%/CF25% increased by 9.78% compared with CNT2%/CF15%.

### 3.4. Mechanical Properties of the PC and ABS Composites

PC has good physical properties because it has one more aromatic benzene ring (C_6_H_6_) compared with ABS. In addition, on the molecular level, ABS is stretched, making it more flexible than PC and more impact-resistant [16].

### 3.5. Comparison of EMI Shielding Effectiveness

**Figure 4:**
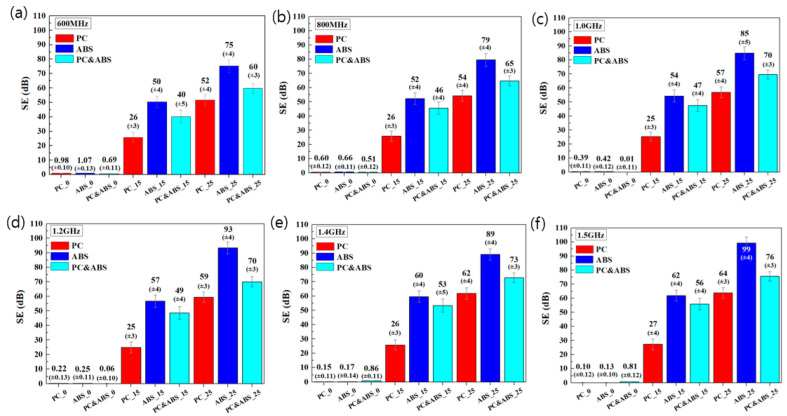
EMI shielding effectiveness of different materials, with respect to the electromagnetic wave frequencies of (**a**) 600 MHz, (**b**) 800 MHz, (**c**) 1.0 GHz, (**d**) 1.2 GHz, (**e**) 1.4 GHz, and (**f**) 1.5 GHz.

The graphs in Figure 4 show the EMI shielding effectiveness of the different composites for electromagnetic wave frequencies in the 0.6–1.5 GHz range. The results in the figure suggest the EMI shielding effectiveness order ABS > PC and ABS > PC. The EMI shielding effectiveness of the PC and ABS specimens was, on average, 39.81% higher compared with the PC specimens, while that of the ABS specimens was 21.57% higher compared with the PC and ABS specimens. As the CF content increased, the EMI shielding effectiveness also increased. CNT2%/CF15% increased by 42.93 dB on average compared with pure specimens, while CNT2%/CF25% increased by 27.80 dB compared with CNT2%/CF15%. In other words, the EMI shielding effectiveness decreases with increasing PC content, concordant with previous reports [17,18]. When PC or ABS were mixed with carbon materials in an internal mixer, the dispersion of the carbon materials was more effective for relatively low-viscosity ABS [19].

As a result, the EMI shielding effectiveness improved, owing to stronger networks between the ABS resin and carbon materials. In addition, the glass transition temperatures are 147 °C for PC and 105 °C for ABS, and it is believed that ABS, which has a relatively low glass transition temperature, softens faster than PC; thus, mixing with carbon materials is more effective [20,21].

### 3.6. Comparison of Surface Resistivity

**Figure 5:**
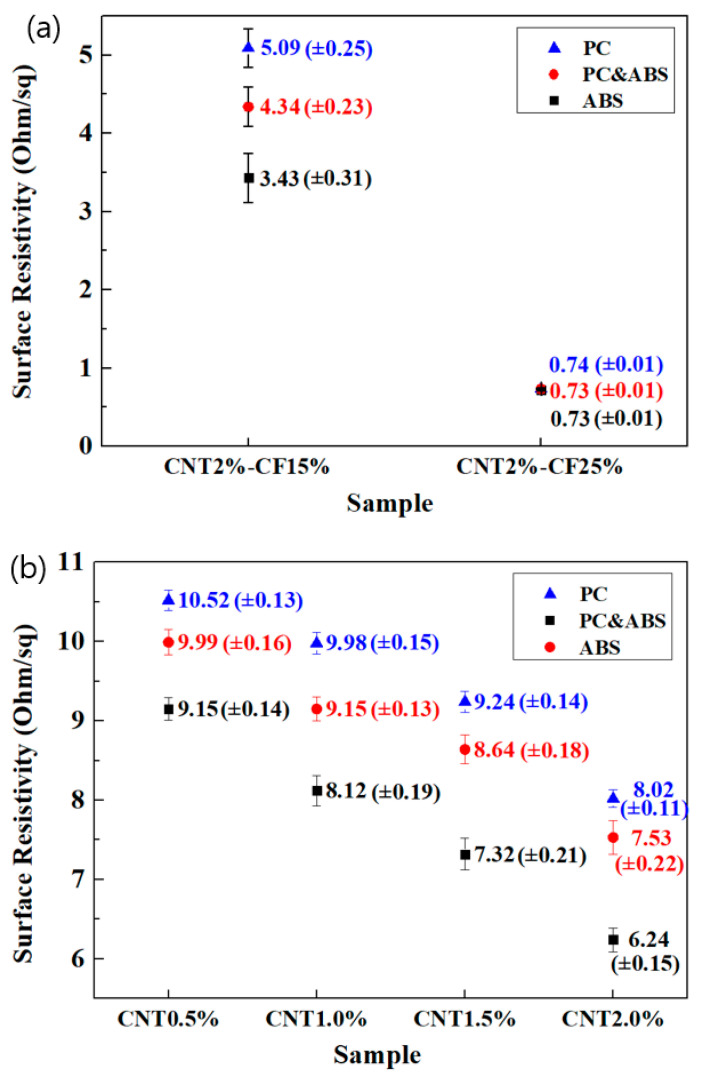
Comparison of surface resistivity according to (**a**) CF and (**b**) CNT content.

The surface resistivity was measured using the four-probe method, in accordance with the ISO 9001 standard. CNT2%/CF15% exhibited resistivity in the order PC > PC and ABS > ABS. As shown in Figure 5a, the surface resistivity of the PC and ABS composites increased by 21.84% on average compared with that of the ABS composites, while the strength of the PC specimens increased by 14.87% compared with that of the PC and ABS specimens. For CNT2%/CF25%, the surface resistivity approached zero as the CF content increased to 25%. In Figure 5b, as CNT content increased from 0.5% to 2.0%, the surface resistivity showed a decreasing trend. As the CF and CNT content increased, the networks inside the specimens became more inter-connected, increasing the specimens’ electrical conductivity and decreasing their surface resistivity. Therefore, the prepared ABS composites exhibited higher electrical conductivity than the prepared PC composites, and the EMI shielding effectiveness also increased owing to the internal network, according to the increase in the electrical conductivity.

### 3.7. SEM-Based Comparison

**Figure 6:**
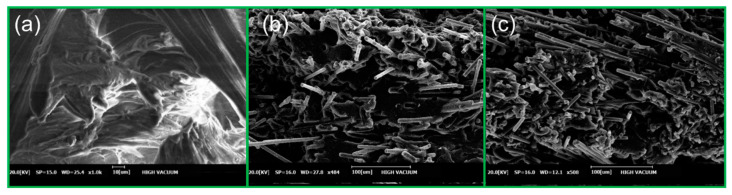
SEM images of the fracture surfaces of the (**a**) pure PC and ABS specimen, (**b**) PC and ABS_ CNT2%/CF15% specimen, and (**c**) PC and ABS_CNT2%/CF25% specimen.

Figure 6 shows the SEM images of the rupture plane, for flexural-strength tests. As Figure 6a reveals, there were no strengthening materials that could strengthen the bond between the PC and ABS resins in the specimens. On the other hand, the SEM images in Figure 6b,c show that chopped CF was scattered in several directions within the PC and ABS resins. Thus, it is believed that the mechanical properties of PC and ABS (tensile strength, flexural strength, and impact strength) are better, and the chopped CF combined with the PC and ABS resins forms networks and increases the specimens’ strength, compared with pure resin specimens.

## 4. Conclusions

In this study, PC and ABS resins are presented as thermoplastic plastics that can lessen the difficulty of processing conventional metal or thermosetting plastics into highly curved exterior covers, have excellent mechanical strength and can function as electromagnetic wave shielding, protecting workers. In addition, in the case of thermoplastic resin, the material was intended to maximize the advantage of being able to reprocess and use even when parts are damaged by applying heat, and to be lighter than existing metals. After adding chopped CFs and CNTs to PC and ABS resins (thermoplastic), the tensile, flexural, impact strength, antistatic performance (characterized by surface resistivity measurements), and the EMI shielding effectiveness were confirmed by testing. Except for the pure PC and ABS specimens, the experiments were carried out by adding 2 wt% of MWCNTs, which is optimal for reducing surface resistivity, and 5, 15, and 25 wt% of chopped CF to prepare the specimens. When the chopped CF content was increased by 25 wt% compared with pure resins, the mechanical flexural, impact, and tensile strengths increased by about 61.09%, 21.44%, 63.56%, respectively, and the SE value (corresponding to the EMI shielding effectiveness) also increased by 70.73 dB significantly. The total average mechanical properties of PC and ABS composites increased by 24.35% compared with that of ABS composites, while that of PC composites increased by 32.86% compared to that of PC and ABS composites. The EMI shielding effectiveness of the PC and ABS composites was increased by 39.81% compared with the PC specimens, while that of the ABS specimens was increased by 21.57% compared with the PC and ABS composites. It was confirmed that the antistatic performance also increased to nearly zero surface resistivity; at the beginning of the study, it was determined that the surface resistivity was minimized when CNTs or CB (carbon black) were added to nylon 66 at 2 wt%. Based on this result, the CNT content was confirmed to be 2 wt%. When chopped CF and MWCNTs were added to the prepared PC and ABS resins, the surface resistivity decreased with increasing content. Therefore, it was confirmed that CFs and CNTs affected the surface resistivity. As the surface resistivity decreased, the electrical conductivity increased, and it was judged to be effective for the improvement of the specimens’ antistatic performance. In the case of metal or thermosetting resin, it has been widely used as an exterior material for a long time until now, but recently, there has been a limiting factor of processability when considering uses in highly curved electronic parts. With a focus on this point, if thermoplastics can be applied to exterior materials, it will likely be used as a material for future electronic parts. The present study contributed to the development of thermoplastic carbon composite materials focusing on the mechanical strength, EMI shielding effectiveness, and antistatic performance for protecting workers from the harmful effects of electromagnetic waves and static electricity. The materials discussed in the present study are likely to become components of novel products.

## Figures and Tables

**Table 1:** Contents of CFs and CNTs added to ABS and PC.

Matrix	Reinforcement	#1	#2	#3	#4
**PC and ABS**	**CNT**	0 wt%	-	2 wt%	2 wt%
	**CF**	0 wt%	-	15 wt%	25 wt%
**ABS**	**CNT**	0 wt%	-	2 wt%	2 wt%
	**CF**	0 wt%	-	15 wt%	25 wt%
**PC**	**CNT**	0 wt%	2 wt%	2 wt%	2 wt%
	**CF**	0 wt%	5 wt%	15 wt%	25 wt%

**Table 2:** Temperature conditions of PC and ABS Resin.

	Melting Point	Internal Mixer	Hot Press
**PC and ABS**	210~300 °C	240 °C	230 °C
**ABS**	210~240 °C	200 °C	180 °C
**PC**	265~300 °C	240 °C	230 °C

## Data Availability

Not applicable.
